# Intrabodies against the Polysialyltransferases ST8SiaII and ST8SiaIV inhibit Polysialylation of NCAM in rhabdomyosarcoma tumor cells

**DOI:** 10.1186/s12896-017-0360-7

**Published:** 2017-05-12

**Authors:** Stefan Somplatzki, Martina Mühlenhoff, Andrea Kröger, Rita Gerardy-Schahn, Thomas Böldicke

**Affiliations:** 1 Helmholtz Centre for Infection Research, Structural and Functional Protein Research, Inhoffenstraße 7, D-38124 Braunschweig, Germany; 2 Helmholtz Centre for Infection Research, Group Innate Immunity and Infection, Inhoffenstraße 7, D-38124 Braunschweig, Germany; 3 Institute of Cellular Chemistry, Hannover Medical School, D-30625 Hannover, Germany

**Keywords:** Neural cell adhesion molecule, Polysialyltransferases, ER intrabodies, Polysialic acid cell surface expression, Xenograft tumor mouse model

## Abstract

**Background:**

Polysialic acid (polySia) is a carbohydrate modification of the neural cell adhesion molecule (NCAM), which is implicated in neural differentiation and plays an important role in tumor development and metastasis. Polysialylation of NCAM is mediated by two Golgi-resident polysialyltransferases (polyST) ST8SiaII and ST8SiaIV. Intracellular antibodies (intrabodies; IB) expressed inside the ER and retaining proteins passing the ER such as cell surface receptors or secretory proteins provide an efficient means of protein knockdown. To inhibit the function of ST8SiaII and ST8SiaIV specific ER IBs were generated starting from two corresponding hybridoma clones. Both IBs αST8SiaII-IB and αST8SiaIV-IB were constructed in the scFv format and their functions characterized in vitro and in vivo.

**Results:**

IBs directed against the polySTs prevented the translocation of the enzymes from the ER to the Golgi-apparatus. Co-immunoprecipitation of ST8SiaII and ST8SiaIV with the corresponding IBs confirmed the intracellular interaction with their cognate antigens. In CHO cells overexpressing ST8SiaII and ST8SiaIV, respectively, the transfection with αST8SiaII-IB or αST8SiaIV-IB inhibited significantly the cell surface expression of polysialylated NCAM. Furthermore stable expression of ST8SiaII-IB, ST8SiaIV-IB and luciferase in the rhabdomyosarcoma cell line TE671 reduced cell surface expression of polySia and delayed tumor growth if cells were xenografted into C57BL/6 J RAG-2 mice.

**Conclusion:**

Data obtained strongly indicate that αST8SiaII-IB and αST8SiaIV-IB are promising experimental tools to analyze the individual role of the two enzymes during brain development and during migration and proliferation of tumor cells.

**Electronic supplementary material:**

The online version of this article (doi:10.1186/s12896-017-0360-7) contains supplementary material, which is available to authorized users.

## Background

The neural cell adhesion molecule (NCAM) is an archetypal member of the family of Ig-domain containing adhesion molecules [1]. NCAM is involved in a number of cell interactions between neurons, neurons and glial cells, neuronal processes and muscle cells, and certain immune cells [2]. A unique feature of NCAM is the existence in two glycoforms, which, due to their prominence in either embryonic or adult tissue, were denominated embryonic- and adult-NCAM [3]. The difference between the two protein forms is the regiospecific addition of polySia onto two *N*-glycans located in 5^th^ Ig-like domain in embryonic-NCAM [4]. PolySia is a homopolymer of the acidic nonasugar sialic acid (Sia) with roughly 100 monomers linked alpha-2,8-glycosidically [5]. Presence of polySia on NCAM has been demonstrated to invert the adhesive functions of the molecule into functions that promote cellular motility and plasticity [6]. Importantly, parts of the plasticity promoting functions of the polySia decorated NCAM (henceforth called polySia-NCAM) attribute the size and negative charge of the polySia shell, which prevents tight cellular interactions and thus globally impedes adhesion processes as well as NCAM dependent cell interactions [7]. With the maturation of tissue (in particular nervous tissue) polySia expression is progressively down regulated and in adulthood has virtually disappeared from peripheral tissues and is detected mainly in different regions of the nervous system [8, 9]. Polysialylation of NCAM is mediated by two Golgi-resident enzymes, the polysialyltransferases (polySTs) ST8SiaII [10, 11] and ST8SiaIV [12, 13], exhibiting overlapping but distinct expression patterns. Each individual enzyme is able to synthesize polySia on NCAM [14]. Thus, selective knockout of only one polyST gene is not sufficient to fully abrogate polySia synthesis in settings where both enzymes are expressed.

Complete abrogation of polySia synthesis in *St8Sia2* ^*−/−*^ *St8Sia4* ^*−/−*^ double-knockout mice causes a postnatal lethal phenotype [15]. PolySTs are crucial for brain development [16]. Interestingly early death in the double-knockout mice is caused by generalized defects also in peripheral organs [15]. Importantly, the most drastic defects observed in *St8Sia2* ^*−/−*^ *St8Sia4* ^*−/−*^ double-knockout mice were selectively rescued by additional depletion of *Ncam*, demonstrating that polySia is essential in steering NCAM interactions in vivo [15]. On the contrary single knockout mice (*ST8siaII* ^*−/−*^ and *ST8siaIV* ^*−/−*^) showed only distinct deficits in histological, electrophysiological and behavioral analyses and thus confirmed that each gene product can at least partially compensates for the absence of the other [17, 18].

### PolySia-NCAM and tumor development

Besides its function in development polySia-NCAM represents a marker in a number of neuroectodermal and neuroendocrine tumors [19, 20]. In polySia-NCAM positive tumors the carbohydrate has been demonstrated to positively impact tumor growth [21–24] and metastasis [25–28]. Endosialidases are phage born enzymes that recognize and degrade polySia with pronounced specificity [29]. To study the role of polySia during tumor progression, knockdown experiments have been carried out in which polySia specific endosialidase were injected [27]. However, endosialidases are large enzymes with restricted penetration into tumor nodules [27] and systemic application of these kinetically stabilized enzymes (the proteins have theoretical half-live times of >100 years) may be followed by long term complications that based on current research cannot be calculated. Taken together, it can be stated, that endosialidases, though attractive, are far away from clinical application. To bypass these limitations and to avoid off-target effects we demonstrate in this study that polySia synthesis can be knocked down by means of intrabody technology.

**Intracellular antibodies (intrabodies)** are very potent molecules for long lasting specific knockdown of proteins. The advantages of IBs are high specificity, the possibility to inhibit post translational modifications [30, 31], and the chance to generate transgenic intrabody mouse models [32–34]. New methods like CRISPR/Cas9 or Talen will be further developed and support the generation of transgenic mice. Moreover, IBs have been proven very useful in cases where the most often used gene-silencing method RNAi produced off target effects [35, 36].

IBs are antibody fragments containing the antigen binding domains, typically in single-chain variable fragment (scFv) format or single domain format. IBs can be targeted to the cytosol, the nucleus, or the ER. Targeting IBs to the ER has proven to be most promising, because ER-resident chaperones and disulfide isomerases ensure correct folding and the formation of essential disulfide bridges, respectively. Due to the lack of these factors in the cytoplasm, IBs in the scFv format were found to be not stable in this compartment [37, 38]. However, a number of techniques has been developed to select functional cytosolic scFv’s [39]. Most successful is the expression of single domain antibodies [39, 40]. Indeed, today the number of cytosolic single domain antibodies is similar to the number of specific ER-IBs.

ER-IBs can be generated from in vitro display systems [41] or by starting from a hybridoma clone [42]. Protein knockdown mediated by ER-IB is based on retention of the formed antigen-antibody complex inside the ER via the ER-retention sequence KDEL fused to the C-terminus of the intrabody [43]. So far, ER-IBs have been elicited mostly against cell surface [39] and two secretory proteins [44, 45]. The technology is, however, also applicable to intracellular proteins as demonstrated by two recent studies, which generated functional knockdowns of TLR9 and sec61 by preventing their recruitment to endosomes by intrabody-mediated retention in the ER [46, 47].

Here we describe the generation of two ER-IBs against the Golgi-localized polySTs, ST8SiaII and ST8SiaIV. The newly generated IBs impeded the cellular polysialylation machinery resulting in reduced polySia-NCAM levels in rhamdyomasarcoma tumor cells. In a xenograft mouse model the metastatic potential of the human rhabdomyosarcoma cell line TE671, which due to the expression of both polySTs is highly positive for polySia-NCAM, could be significantly reduced. Combined application of both ER IBs resulted in a clear delay in metastasis. This highlights the efficacy of ER IBs as novel tools to interfere with Golgi localized glycosyltransferases and to study the impact of aberrant glycosylation in cancer biology.

## Results

### Construction and expression of IBs against the polysialyltransferases ST8SiaII and ST8SiaIV

**Fig. 1:**
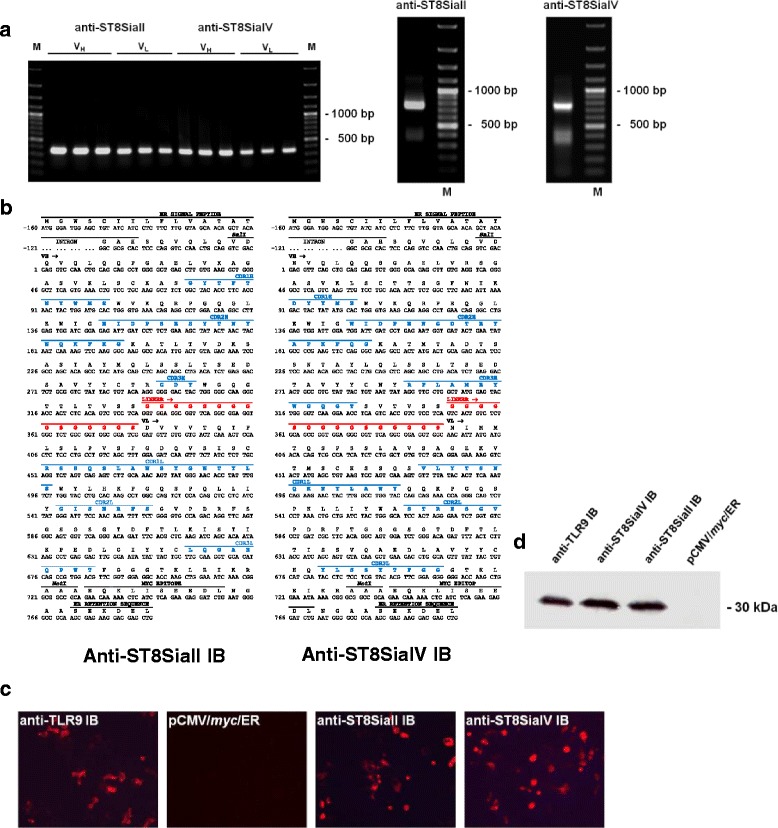
Assembly and expression of ER IBs recognizing ST8SiaII and ST8SiaIV. **a** PCR amplification of the variable domains VH and VL of anti-ST8SiaII and anti-ST8SiaiIV scFv fragments (left side) and assembly of the corresponding scFvs (right side). 20 μl of the PCR products were analyzed on a 1% agarose gel (M = 100 bp ladder). **b** Primary sequence of anti-ST8SiaII and anti-ST8SiaIV intrabody. Shown are the coding (lower lane) and amino acid sequence (upper lane) of anti-ST8SiaII-IB and anti-ST8SiaIV-IB including the ER signal peptide, the *myc* epitope and the ER retention sequence. The complementary-determing regions (CDR1-CDR3) of the variable domains of the heavy and light chain are printed in blue letters. The synthetic linker, shown in red letters, localized between the VH and VL domains was introduced by assembly PCR. **c** Immunofluorescence analysis by microscopy of permeabilized and fixed HEK293 cells transiently transfected with IBs. Intracellular expression was stained in red. Positive control: anti-TLR9 IB, negative control: HEK 293 cells transiently transfected with the empty expression plasmid pCMV/myc/ER. **d** Immunoblot analysis, Expression of anti-ST8SiaII-IB and anti-ST8SiaIV-IB visualized with peroxidase labelled secondary antibody. Sample volume: 10 μl of 100 μl cell lysat from 10^6^ cells transiently transfected for 48 h with the intrabody DNA in a 6-well microtiter plate

ER IBs against the polySTs ST8SiaII and –IV (αST8SiaII-IB and αST8SiaIV-IB, respectively) were generated on the basis of the variable domains of the monoclonal antibodies (mAb) 3167 and 3175, which are directed against ST8SiaII and ST8SiaIV, respectively. The corresponding DNA sequences were amplified by RT-PCR on mRNA isolated from the respective hybridoma clones. The sequence stretches encoding the variable regions of heavy (V_H_) and light chain (V_L_) encompassed 351 bp and 353 bp for αST8SiaII-IB and 363 bp and 353 bp for αST8SiaIV-IB. The DNA fragments encoding V_H_ and V_L_ were connected through the sequence stretch (Gly_4_ Ser)_3._ The resulting scFv constructs comprised 749 bp for αST8SiaII-IB and 761 bp for αST8SiaIV-IB (Fig. 1a). Cloning into the vector pCMV/*myc*/ER resulted in plasmids that encoded scFv’s with an N-terminal signal peptide and a C-terminal myc-tag followed by an ER retention signal (Fig. 1b). Notably, the original sequences encoding the variable domains were obtained by a method based on PCR amplification of adaptor ligated cDNA [42]. Therefore an adaptor sequence is ligated to double stranded cDNA’s of both antibodies.

Subsequent PCR with primers binding to the beginning of the adapter sequence and the constant domain of IgG1 respectively leads to amplification of the adaptor, leader, variable domain and part of the constant IgG1 domain. This technique delivers the correct sequences and prevents mismatches which might occur if the variable domains are amplified by consensus primers [48, 49]. Interestingly, the sequence of the CDR3H region of ST8SiaII-IB is very short comprising only 3 amino acids.

After transient transfection of HEK293 cells, expression of IBs was demonstrated by immunofluorescence staining (Fig. 1c) and Western blot analysis with the anti-myc mAb 9E10 (Fig. 1d). Immunoblotting revealed an apparent molecular mass of approximately 30 kDa, which is characteristic for ER-IBs in the scFv format (Fig. 1d) [50].

### Binding of IBs to polysialyltransferases ST8SiaII and ST8SiaIV

**Fig. 2:**
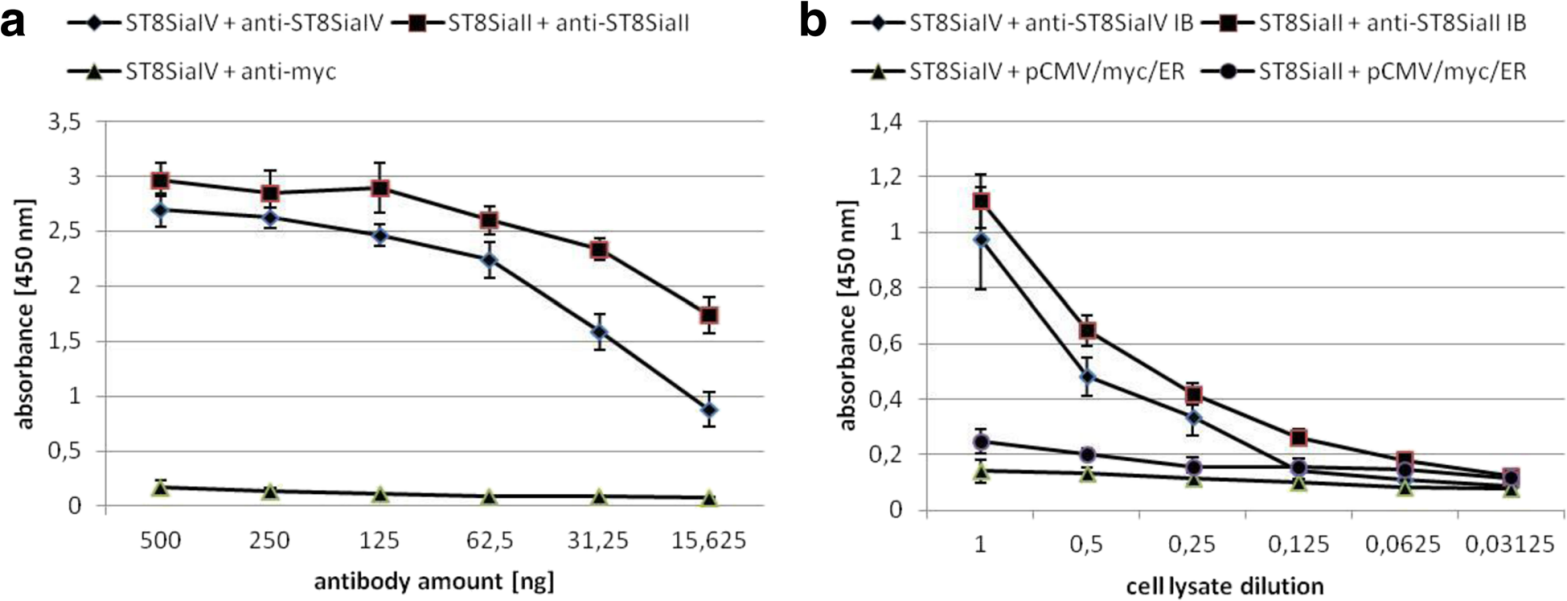
Binding of anti-ST8SiaII-IB and anti-ST8SiaIV-IB to their antigens in ELISA. **a** 50 ng purified ST8SiaII and ST8SiaIV in 50 μl 0.2 M sodium phosphate puffer was immobilized on MaxiSorb™ polystyrene assay plates (Nunc) as indicated. Serial dilutions of purified original anti-ST8SiaII and anti-ST8SiaIV mAbs 3167 and 3175, respectively, were applied in 100 μl PBS. Negative control: ST8SiaIV incubated with anti-myc antibody. **b** Serial dilutions of 100 μl cell lysates of 10^6^ HEK293 cells transiently transfected with anti-ST8SiaII-IB expression plasmid or anti-ST8SiaIV-IB expression plasmid for 48 h in a 6 well microtiter plate were incubated in different serial dilutions in 100 μl PBS with immobilized purified ST8SiaII or ST8SiaIV. Negative controls: Cell lysates transfected with pCMV/myc/ER. Result of 3 independent experiments. Bars demonstrate standard deviation calculated from the mean values

**Fig. 3:**
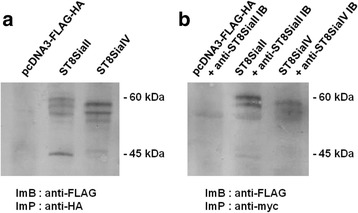
Intracellular Binding of anti-ST8SiaII-IB and anti-ST8SiaIV-IB to their antigens. **a** control, immunoprecipitation of FLAG-HA tagged ST8SiaII and FLAG-HA tagged ST8SiaIV transiently transfected in 10^6^ HEK293 cells for 48 h in a 6-well microtitre plate. After lysis in 100 μl lysisbuffer and immunoprecipitation the different glycosylated forms were analyzed by immunoblotting. Negative control: transfection with empty vector pcDNA3-FLAG-HA. **b** Co-IP of HEK 293 cells cotransfected with FLAG-HA tagged ST8SiaII and ST8SiaII-IB or FLAG-HA tagged ST8SiaIV and ST8SiaIV-IB. Negative control: Co-IP of HEK293 cells cotransfected with pcDNA3-FLAG-HA and anti-ST8SiaII-IB expressionplasmid. ImB: Immunoblot; ImP: Immunoprecipitation. Sample volume is 12 μl from total 25 μl immunoprecipitat. The experiment was done 2 times, shown is a representative example

To confirm that the newly generated ER IBs maintained the antigen binding activity of the original mAbs, we performed an ELISA. Immobilized FLAG-HA tagged ST8SiaII and ST8SiaIV were incubated with the original mAbs 3167 and 3175 (Fig 2a) as well as with serial dilutions of cell lysates from HEK293 cells, which had been transiently transfected with either one of the intrabody expression plasmids or with empty vector (Fig. 2b). (Additional file 1). Compared to cell lysates from empty vector transfected HEK293 cells, significant antigen binding was detected for intrabody containing HEK239 lysates. Consistent with this, the formation of intracellular intrabody-antigen complexes was demonstrated (Fig. 3) by co-immunoprecipitation. Therefore, HEK239 cells were co-transfected with plasmids driving the expression of the respective Flag-HA-tagged polyST and the corresponding myc-tagged intrabody. Interaction was demonstrated by capturing the IBs via their C-terminal myc-epitope. An efficient co-immunoprecipitation of the respective polySTs was demonstrated by Western blot analysis with anti-Flag antibody (Fig. 3b). Co-immunoprecipitation resulted in the same band pattern as direct immunoprecipitation of the enzymes by an anti-Flag antibody (Fig. 3a). As shown earlier, ST8SiaII and ST8SiaIV contain several N-glycosylation sites and in addition to the fully glycosylated variants with apparent molecular masses of 60 kDa and 55 kDa, respectively, glycoforms with fewer N-glycans and increased electrophoretic mobility were found [51].

### αST8SiaII-IB and αST8SiaIV-IB mediate ER-retention of the corresponding polysialyltransferase

To investigate if anti-polyST IBs provoke ER-retention of their cognate polyST, we analyzed the subcellular localization of polySTs in the presence and absence of the IBs. For this approach, we made use of stably transfected CHO cells derived from the polyST-deficient mutant 2A10 [52]. Clone CHO-2A10 + 500 stably expresses Flag-HA-tagged ST8SiaII and clone CHO-2A10 + 418 Flag-HA-tagged ST8SiaIV [51]. Both cell lines were transiently transfected with each of the intrabody expression plasmids (or empty vector) and the expressed IBs and polySTs were visualized by immunofluorescence analysis.

**Fig. 4:**
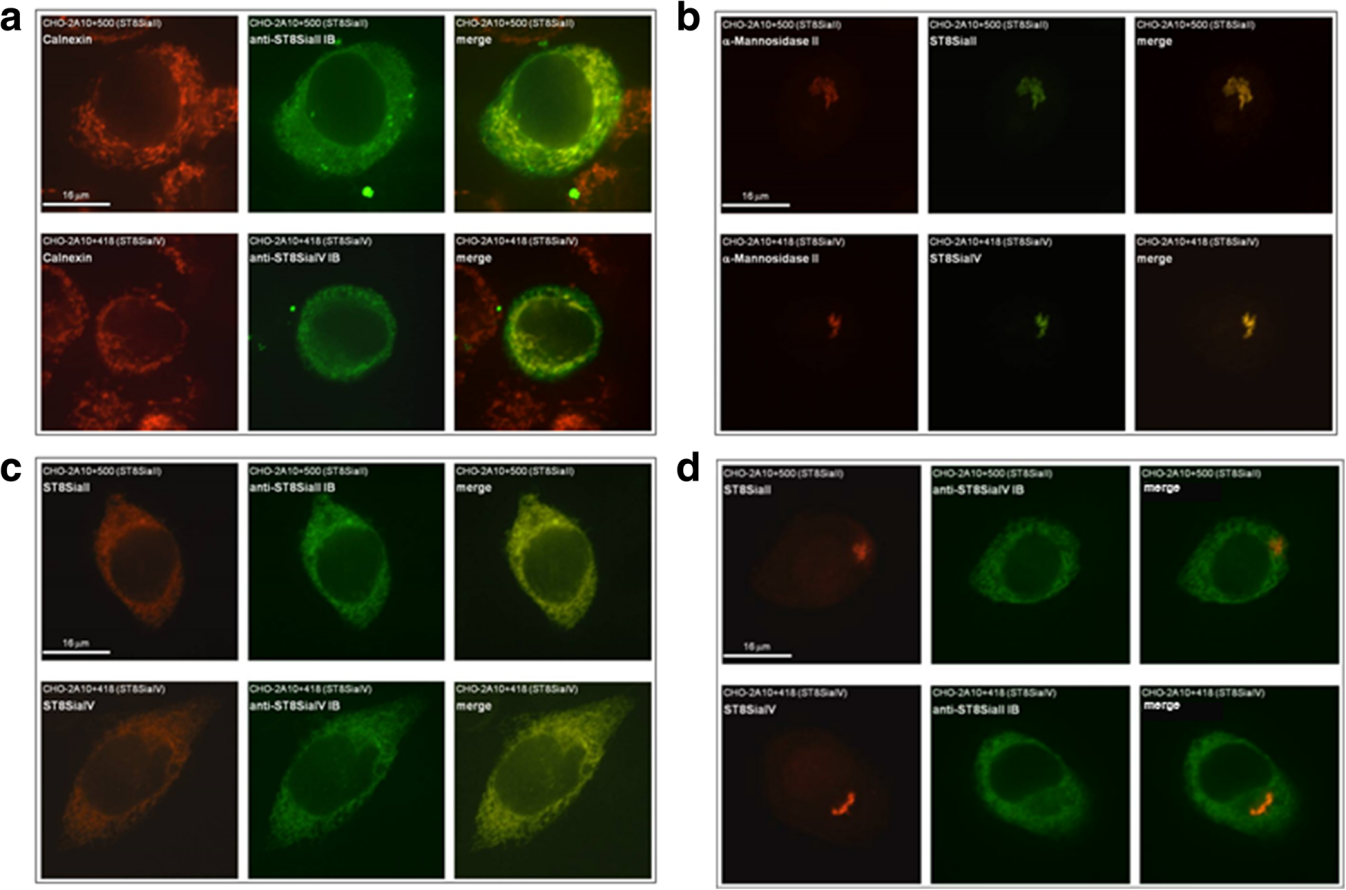
Anti-ST8SiaII-IB and anti-ST8SiaIV-IB mediates retention of ST8SiaII and ST8SiaIV inside the ER. 10^6^ recombinant CHO cells transiently transfected for 48 h with the corresponding intrabody DNA or unspecific intrabody DNA in a 6-well microtiter plate were analyzed by immunofluorescence. **a** Colocalisation of IBs with ER marker Calnexin. Recombinant CHO cells expressing ST8SiaII (CHO-2A10 + 500) were transfected with anti-ST8SiaII-IB expression plasmid (upper line) or CHO cells expressing ST8SiaIV (CHO-2A10 + 418) were transfected with anti-ST8SiaIV-IB expression plasmid (lower line). ER resident marker Calnexin was stained red. IBs were labelled green. **b** colocalisation of ST8SiaII and ST8SiaIV in non transfected CHO-2A 10 + 500 cells (upper line) and in non transfected CHO-2A10 + 418 cells (lower line) with Golgi marker α-mannose II. Golgi marker α-mannose II was stained red. Polysialyltransferases were labeled green. **c** ST8SiaII and anti-ST8SiaII-IB or ST8SiaIV and anti-ST8SiaIV-IB were stained in anti-ST8SiaII-IB or ST8SiaIV-IB transfected CHO-2A10 + 500 cells or CHO-2A10 + 418 cells. In this case the polysialyltransferases were stained red **d** negative controls: Staining of the polysialyltransferases and IBs in the CHO cells transfected with the unspecific IBs. The experiment was done 3 times. Shown is a characteristic staining

Co-localization of the IBs with the ER marker calnexin verified the presence of the IBs in the ER (Fig. 4a). In the absence of IBs, the anti-Flag signals (detection of polySTs) clearly co-localized with the Golgi marker α-mannosidase II and thus indicated Golgi localization of polySTs (Fig. 4b). However, upon intrabody expression, the targeted polySTs co-localized with the intrabody in the ER, thus indicating efficient ER retention (Fig. 4c). Of importance, expression of αST8SiaII-IB affected the localization of ST8SiaII (Fig. 4c, upper panel) but not of ST8SiaIV (Fig. 4d lower panel). Vice versa, expression of αST8SiaIV-IB resulted in ER-retention of ST8SiaIV (Fig. 4c lower panel) but not of ST8SiaII (Fig. 4d, upper panel), underlining the specificity of the IBs.

### Expression of αST8SiaII-IB and αST8SiaIV-IB reduces polySia expression in CHO cells

**Fig. 5:**
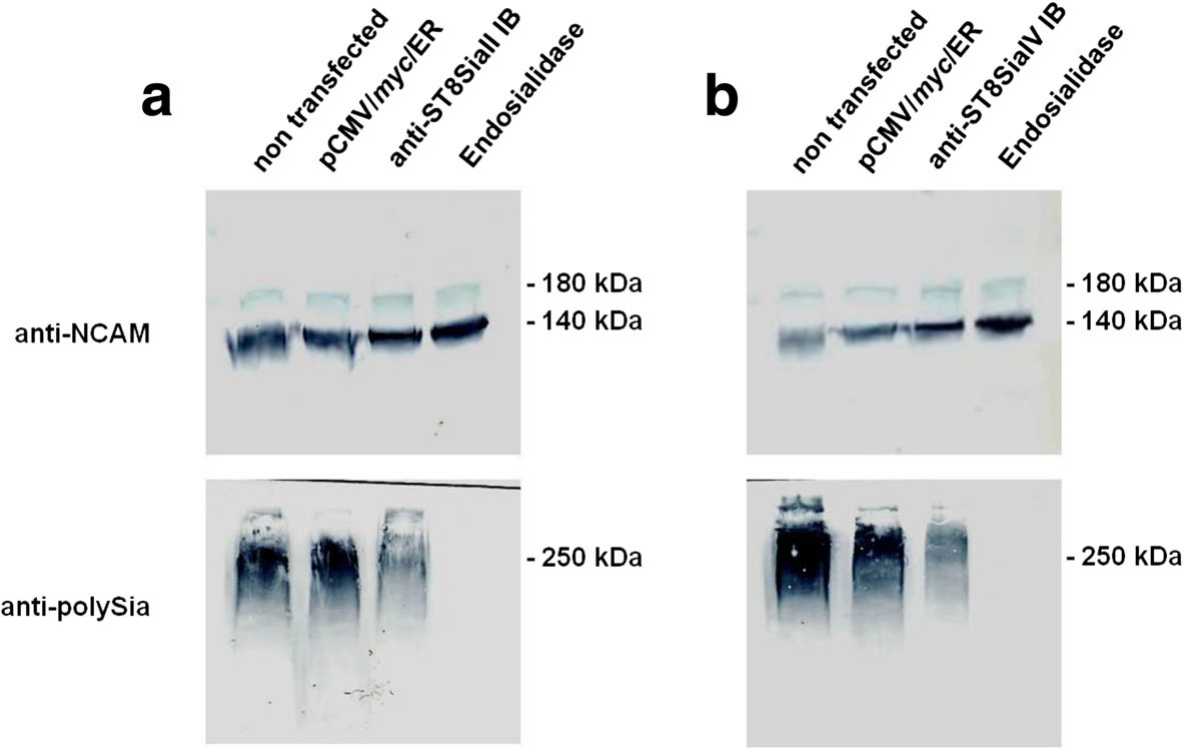
Reduced expression of polySia in recombinant CHO cells mediated by anti-ST8SiaII and anti-ST8SiaIV IBs. **a** Immunoblot analysis of expressed NCAM and polySia of NCAM in CHO-2A10 + 500-cells expressing ST8SiaII transfected with ST8SiaII-IB. Negative control: cells non transfected or transfected with pCMV/myc/ER. Positive control: cell lysat treated with endosialidase. **b** Immunoblot analysis of expressed NCAM and polySia of NCAM in CHO-2A10 + 418-cells expressing ST8SiaIV transfected with ST8SiaIV IB. The experiment was done 3 times. Shown is a characteristic example. Sample volume: 10 μl of 100 μl cell lysat from 10^6^ cells grown for 48 h in a 6-well microtiter plate

To monitor the functional consequences of intrabody-mediated ER-retention of polySTs, the polySia levels were monitored in CHO-2A10 + 500 (expresses ST8SiaII) and CHO-2A10 + 418 cells (expresses ST8SiaIV) before and after transient expression of the respective intrabody. As known from previous studies, the polyST transformed 2A10 cell lines express the two major NCAM isoforms NCAM-140 and NCAM-180 but polysialylation of NCAM isoforms is incomplete in these cells [52]. In keeping with this information, the anti-polySia mAb 735 revealed in both cell lines a broad microheterogenous polySia-NCAM signal centering at an apparent molecular mass of 250 kDa, while staining with mAb H28, specifically recognizing a protein epitope on NCAM, displayed two focused bands that represented polySia-free NCAM-140 and −180 (Fig. 5). Applying these antibodies to intrabody expressing cells, demonstrated a significant decrease in the polySia signal while the NCAM signals remained unchanged. Based on these results it can be stated that the ER-IBs directed against ST8SiaII- and IV are efficient in knocking down the polySia production on NCAM while the NCAM protein itself remained untouched.

### PolySia knockdown in human rhabdomyosarcoma cells stably expressing αST8SiaII-IB and αST8SiaIV-IB

**Fig. 6:**
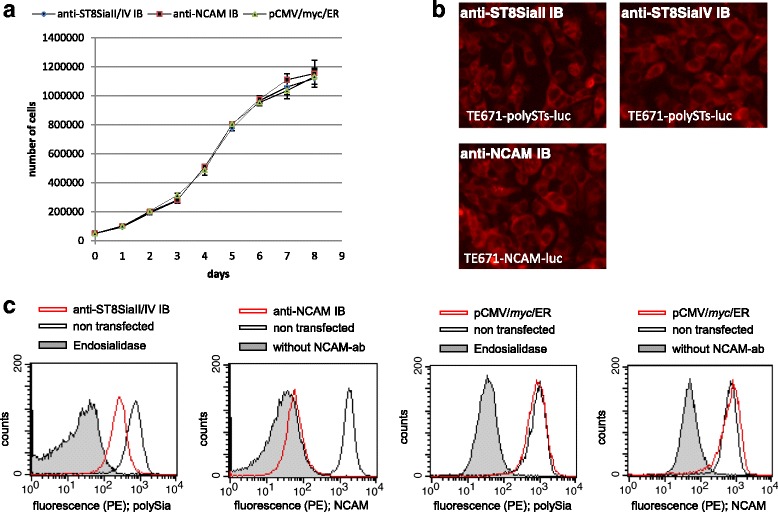
PolySia cell surface expression is inhibited in TE671 rhabdomyosarcoma cells expressing α ST8 Sia II and α ST8 Sia IV intrabody. **a** Proliferation assay of 5 x 10^4^ TE671 cells stable expressing αST8SiaII-IB and αST8SiaIV-IB, anti-NCAM IB or empty vector cultured in wells of a 6-well microtiter plate over a time period of 8 days. The number of living cells was determined after staining with trypan blue using a Rosenthal-Fuchs counting chamber. Starting material was 5 × 10^4^ cells. The proliferation assay was done 3 times, bars demonstrate standard deviation calculated from the mean values. **b** Immunofluorescence analysis of anti-ST8SiaII-IB, anti-ST8SiaIV-IB and anti-NCAM intrabody expression. 10^6^ recombinant CHO cells transiently transfected for 48 h with the anti-PolySTs intrabodies or anti-NCAM intrabody in a 6-well microtiter plate were analyzed by immunofluorescence. Anti-ST8SiaII, Anti-ST8SiaIV-IB and anti-NCAM intrabody were detected in red. **c** Flow cytometric detection of polySia and NCAM cell surface expression. Stable transfected cell lines are shown with red lines. Negative controls are non-transfected cells (grey line) and cells only incubated with the secondary antibody (grey area). In the case of detection of polySia negative controls are cells treated with endosialidase (grey area). 10^4^ cells in 300 μl PBS containing 2% FCS and 10 μg/ml propidiumiodide were measured. The flow cytometric analysis was performed 4 times. Shown is characteristic cell surface staining of NCAM and polySia from one experiment

High polySia expression in the human rhabdomyosarcoma cell line TE671 is due to the expression of both polySTs [53]. Interrogating if combined application of the newly generated anti-polyST IBs can reduce polySia expression rhabdomyosarcoma cells were double transfected with plasmids driving the expression of αST8SiaII-IB and αST8SiaIV-IB. As it was our goal to use transformants also in in vivo experiments (see next paragraph), stable intrabody-expressing cell lines were generated that simultaneously expressed luciferase as a reporter protein. Moreover, for control reasons we also generated an NCAM-knockdown variant in TE671 by use of the previously described αNCAM-IB plasmid [42]. Three stably transfected TE671 clones were selected. (1) TE671-polySTs-luc: TE671 cells expressing αST8SiaII-IB, αST8SiaIV-IB and luciferase. (2) TE671-NCAM-luc: TE671 cells expressing α-NCAM-IB and luciferase. (3) TE671-control: TE671 cells expressing luciferase and transfected with the empty vector pCMVmycER. All clones were analyzed for proliferation, intrabody expression and intrabody functionality. Identical proliferation rates were measured under standard cell culture conditions (Fig. 6a) and immunofluorescence staining demonstrated comparable expression levels of the two anti-polyST IBs (Fig. 6b). After 14 days in culture, flow cytometric was used to analyze polySia levels in αST8SiaII-IB/αST8SiaIV-IB expressing TE671 cells. As shown in Fig. 6c a significant reduction in the cell surface expression of polySia was found but not of NCAM (data not shown). As expected, application of the αNCAM-IB most efficiently downregulated NCAM as well as polySia surface expression. By contrast, cell surface expression of NCAM and polySia were unaltered in the mock transfected control cell line (Fig. 6c).

### TE671 rhabdomyosarcoma cells expressing anti-polyST IBs show delayed metastasis in a xenograft tumor mouse model

**Fig. 7:**
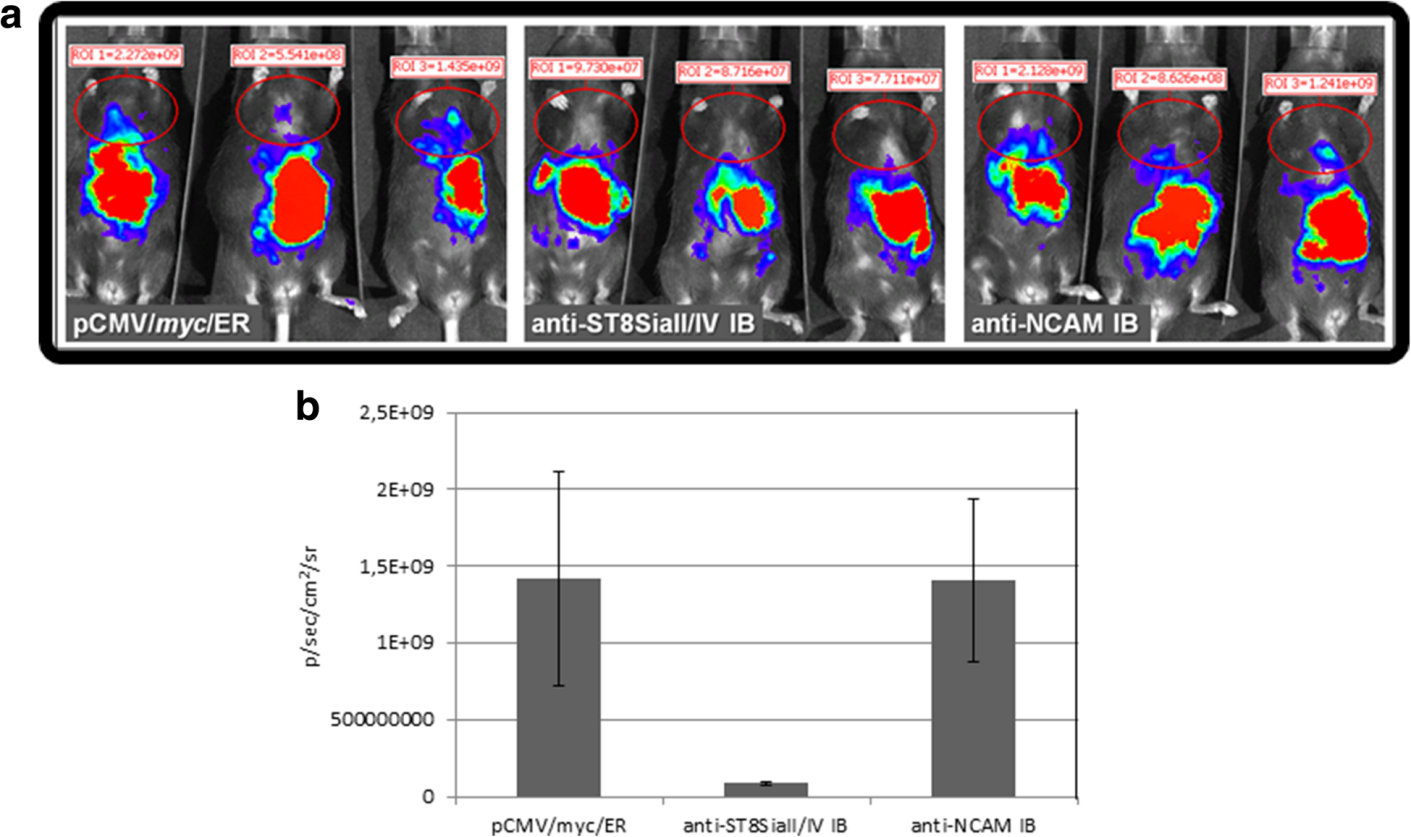
Inhibitory effect of anti-ST8SiaII-IB and anti-ST8SiaIV-IB on metastasis of rhabdyomasarcoma cells after 4 weeks of tumor cell injection in mice. **a** Detection of tumor cells by luminescence. 10^6^ rhabdomyosarcoma cells in 100 μl PBS expressing anti-ST8SiaII-IB and anti-ST8SiaIV-IB or anti-NCAM-IB or as negative control tumor cells stable transfected with the empty vector were injected intraperitoneally into 3 C57BL/6 J RAG-2 mice at a time. Luminiscence was determined at week 4 using in vivo imaging systems (IVIS). Shown are the results as luminiscence signals (p/sec/cm^2^/sr). ROI: region of interest. The red circles shows the metastic tumor cells in the region of lung and liver. **b** Mean values of each group of mice with corresponding standard deviation. Tumor growth was tracked every week over a period of 6 weeks. Complete tumor tracking is shown in supplement data

The isolated clones TE671-polySTs-luc,TE671-NCAM-luc, and TE671-control were next used to investigate metastasis formation in a xenograft tumor mouse model. In this experiment 10^6^ cells were injected intraperitoneally (i.p.) in 20 weeks old C57BL/6 J RAG-2 mice that lack the production of B-, T- and NK-cells. Each cell line was injected into 3 mice. Tumor growth and metastasis formation was monitored over 6 weeks by in vivo imaging of luminescence after i.p. injection of a luciferine solution. Between week 1 and 4, peritoneal tumors grew in all mice. At week 4 lung and/or liver metastases were observed in all mice injected with TE671-control and TE671-NCAM-luc cells. In contrast, in mice injected with TE671-polySTs-luc cells no distant metastasis were detectable at this time point (Fig. 7) (Additional files 2, 3, 4 and 5).

Although all xenografts showed metastatic spread after 4 weeks (Additional file 6: Figure S1 and Additional file 7: Figure S2) our results provide initial evidence that application of anti-polyST IBs delayed the metastatic spread of TE671 rhabdomyosarcoma cells.

## Discussion

PolySia is a developmentally regulated posttranslational modification predominantly found on NCAM [6]. As a regulator of NCAM [54] interactions, polySia is essential for postnatal development and brain wiring [15]. Moreover, polySia represents an oncofetal antigen, which is widely expressed in fetal but not in adult tissues and commonly re-expressed on tumors of neuroectodermal and neuroendocrine origin such as small cell lung carcinoma, neuroblastoma, and rhabdomyosarcoma [21–24, 54]. PolySia-positive tumor cells typically express ST8SiaII and ST8SiaIV [53, 55] and therefore to block polySia synthesis, inhibition of the two enzymes is required to ablate polysialylation.

To overcome problems that might arise from off-target effects and to generate molecules for specific knockdown of each enzyme we selected the intrabody approach. For inhibition of the function of transitory molecules IBs are targeted to the ER where they bind to their antigens very specifically [39]. Consequently, the translocation of the antigen to the final cell compartment is blocked. The IB technology has been successfully used to knockdown targets in vitro and in vivo [32–34, 39]. A recent example of the exquisite specificity of IBs has been provided by the use of anti-TLR IBs [46, 50]. In the current study, anti-polyST IBs were constructed from the variable domains of two monoclonal antibodies (Fig. 1).

Analyzing the function of ST8SiaII-IB and ST8SiaIV-IB using immunoblot analysis (Fig. 5) demonstrated efficient knockdown for each enzyme. The inhibition of polysialylation mediated by the IBs is due to intrabody-polyST complex formation inside the ER. The fact, that polySTs do not reach the Golgi apparatus prevents the enzymes from gaining access to their substrates CMP-sialic acid and NCAM with fully processed N-glycans (Fig. 4c). Although costaining of the IB with the ER marker calnexin (Fig. 4a) and the antigens (Fig.4c) confirmed ER retention, the signal displayed by calnexin was much more coarse and discontinuous than the signal obtained if IBs were displayed with anti-myc antibody and polyST with anti-FLAG antibody. However, this effect is known and has similarly been observed with anti-TLR2 IBs [50].

Importantly, the efficiency of the IBs to modulate the metastatic potential of a rhabdomyosarcoma xenograft (TE671 cells) was investigated in a mouse model. In this study tumor cells stably expressing both anti-polyST IBs were used in comparison to control cells and to cells expressing the recently generated anti-NCAM IB. Although the inhibition of NCAM-polysialylation was not complete in TE671 cells transformed with anti-polyST IBs, the knockdown was sufficient to significantly delay tumor spread (Additional file 6: Figure S1 and Additional file 7: Figure S2). After 6 weeks no inhibitory effect of the IBs could be seen anymore and metastasis was similar as seen with the control tumor cells (Additional file 6: Figure S1 and Additional file 7: Figure S2). The inhibition of metastasis was not complete mainly due to the fact that the knockdown of polysialylation in TE671 cells was not complete. Furthermore it cannot be excluded that tumor cells with integrated IB genes have loss the IB genes after 6 weeks and may overgrow the stable IB expressing cells. This indicates that more experimental work is needed to achieve a complete polySia depletion. A combination of Endo-N treatment with intrabody expression might further reduce polysialylation.

It is known that many factors are involved in metastasis such as lost of cadherin or integrin [64] and cell migration-promoting chemokines. Pro-metastatic factors activate specific, corresponding types of receptors [65]. Our targets were the polySTs and we cannot exclude that metastasis is enhanced after 4 weeks due to other molecules affecting metastasis not targeted by our specific intrabodies.

Other reasons might also be taken into account. One possibility is that polySTs bind to NCAM inside the ER and this interaction interferes with intrabody binding. To interrogate this possibility we performed an ELISA based test. Immobilized NCAM was preincubated with the polySTs, which were then detected with the IBs. In this test system we did not recognize inhibition of IB-binding to the polySTs (data not shown).

Eventually, one has to bear in mind that the developed IBs target enzymes. Since IBs generally knockdown antigens it might well be that low amounts of the active enzymes (below detection level) reach the Golgi and are sufficient to produce significant amounts of polySia-NCAM.

In a similar metastatic mouse model Daniel et al. [24] injected TE671 cells intraperitoneally and removed polySia by co-injection with Endo-N. Analogous to our study ascites formation was decreased and the number of lung or liver metastases reduced. Also in this earlier study the failure to completely block tumor formation was explained by incomplete removal of polySia from tumor cells.

ER intrabodies comprising the C-terminal KDEL retention sequence have been successfully used to inhibit the function of transitory protein. Inhibition was in the range from 50% to 100% [57]. The KDEL sequence leads to retrograde transport from different subcellular Golgi compartments. Transitory proteins can be recycled from an intermediate compartment before the protein has reached the Golgi-complex, from the cis Golgi compartment and even from the trans-Golgi compartment [58]. Recently it was shown that a chimeric sialyltransferase (ST) with a rapamycin binding domain (FRB) binding to FKBP (second rapamycin binding domain) protein containing a C-terminal KDEL sequence lead to retrograde transport from the Golgi to the ER [59]. From which Golgi compartment ST was recycled was not estimated.

Retrievel from an early Golgi complex location was easily shown by Golgi-specific modifications of the *N-* or *0-*linked carbohydrate side chains of transitory proteins [60]. In Fig. 4 it is clearly shown that in the presence of the specific intrabody the enzyme are found exclusively in the ER and not inside the Golgi network. However this is only one time point within the retention process.

Transferring Polysia to NCAM by ST8SiaII and ST8Sia IV occurs in the trans Golgi [61]. However we cannot exclude that the enzyme intrabody complexes are recycled from the trans Golgi and binds to NCAM. If the antibody inhibits binding of the enzyme to NCAM or inactivates the enzyme so that polysialylation cannot occur is not clear. Another possibility that intrabody inhibition is not complete might be that the affinities of the original anti-polySTs are not high enough for complete retention of the antigens inside the ER, although we were not able to identify polySTs in the Golgi compartment in IB expressing cells (Fig. 4c).

To compare the knockdown of ST8SiaII and ST8SiaIV mediated by the anti-polySTs IBs with the knockdown of complete NCAM-polySia we injected tumor cells expressing an anti-NCAM intrabody established recently by us [42]. We wanted to analyse if abrogation of NCAM-polySia at the cell surface would lead to metastasis even if integrins and cadherins and the cadherin associated proteins are not abrogated.

We expected that abrogation of NCAM-polySia on the tumor cell surface would led to metastasis of the cells. Indeed, tumor cells expressing the anti-NCAM intrabody showed metastatic behavior similar as the control tumor cells. This confirms the published data that abolished NCAM on the surface of tumor cells promotes metastasis [56, 66, 67]. Recently it was found that other cell surface proteins as NCAM can be polysialylated (i.e. SynCAM-1, Neuropilin-2, chemokine receptor CCR7 and E-selectin ligand-1) [68–71]. From these proteins CCR7 is expressed on rhabdomyosarcoma cells and is involved in lymph node metastasis [72] We cannot conclude if inhibition of polysialylation of CCR7 by the IBs have also contributed to the delay of metastasis.

Alternatively to the IB approach, a knockout of polySTs can be performed with the CRISPR/Cas9 technology . Recently this approach has been used to show that ST8SiaVI-dependent cell surface polysialylation is essential for endoderm specification [62]. In regard to the CRISPR/Cas9 technology one advantage of the intrabody technology is that the intrabodies can be transfected by viral gene transfer in almost every cell line. On the contrary if one intend to study the function of the polySTs in different cell types new knockout cell lines using CRISPR/Cas9 have to be generated. Furthermore in contrast to the CRISPR/Cas9 technology which often showed off targets effects, such effects have not been seen so far with intrabodies [57, 63].

At the moment the number of ER IBs as well as cytosolic nanobodies is increasing. They are very useful to study the function of proteins in vitro and in vivo. Beyond some IBs have been shown to have therapeutic potential. Among the most promising candidates are those that impact viral infections, brain diseases and cancer [39]. The successful therapeutic application of IBs depends on the development of safe viral vectors and nonviral vectors with high gene transfer efficiency. Current viral vectors for gene therapy are associated with serious safety concerns including insertional mutagenesis [73] and usage of nonviral vectors is limited by low gene transfer efficiency [74]. Furthermore tissue-specific transfer of intrabody genes and therapeutic transgenes in general is important for safe and effective gene therapy. Many viral vectors are not specific for one cell type. By transductional targeting cell-type specific engineered viruses can be transduced specifically [75]. This includes engineering of adaptor proteins containing the targeting ligands, monoclonal antibodies or bispecific antibodies into the virus envelope or changing of the serotype [75]. Another method is transcriptional targeting based on expression of the transgene via tissue-specific promoters [76]. This method is limited by the small number of strong tissue specific promoters. A third approach based on microRNA-regulated viral vectors has been developed [77]. Here artificial microRNA target sites are incorporated into the viral vector serving as targets for a specific microRNA leading to transcript degradation. This technique has the potential to be translated into clinical applications. Furthermore conferring to non viral transfections nanoparticles decorated with cell surface receptor specific nanobodies are working in many examples in vitro and will now start to be tested in vivo [78].

In the future a promising alternative approach might be the application of mRNA [79, 80]. Furthermore it might be possible to combine the intrabody strategy with other strategies which aim to inhibit the function of the polySTs in cancer cells [81, 82]. To the best of our knowledge this is the first description of ER IBs (αST8SiaII-IB and αST8SiaIV-IB) that knockdown Golgi-located enzymes in vitro and in vivo.

## Conclusion

The data indicate that the new IBs are potent tools to study the individual role of each enzyme in cell migration and tumor progression of different tumors. In addition they can be used to get more insight into the role of ST8SiaII and ST8SiaIV on the polysialylation of targets different from NCAM (i.e. SynCAM-1, Neuropilin-2, the chemokine receptor CCR7, and E-selectin ligand-1) [68–71].

## Methods

### Cells and mice

HEK293 cells were obtained from the DSMZ, Braunschweig. TE671 cells were from R. Gerardy-Schahn, MHH, Hannover. Recombinant CHO-2A10 cells expressing N-terminally Flag-HA-tagged murine ST8SiaII (CHO-2A10 + 500) or Flag-HA-tagged hamster ST8SiaIV (CHO-2A10 + 418) were generated as described [51]. Hybridoma cells secreting anti-ST8SiaII IgG1 mAb 3167 and anti-ST8SiaIV IgG1 mAb 3175 were generated by fusing murine X63-Ag8.653 myeloma cells with splenic lymphocytes of immunized BALB/c mice. Immunization was performed with purified Protein A-polyST fusion proteins, which compassed Ser26 to Thr375 of murine ST8SiaII or Arg27 to Gln359 of hamster ST8SiaIV [51]. The obtained mAbs 3167 and 3175 recognize the rodent polySTs used for immunization as well as the corresponding human counterpart. The anti-ST8SiaII mAb does not cross-react with ST8SiaIV and *vice versa* was observed no cross-reactivity of anti-ST8SiaIV mAb with ST8SiaII. C57BL/6 J RAG-2 mice were from A. Kröger (HZI).

### In vitro maintenance of transfected HEK293, CHO or TE671 cells

The HEK 293 cells were transfected with ST8SiaII or ST8SiaVI or cotransfected with the corresponding IBs to demonstrate binding of PolySTs to the intrabodies by Co-IP (Fig. 3). For verifying the binding of the IBs to the PolySTs in Elisa the IBs were also express in the HEK293 cells (Fig. 2).

The recombinant CHO cell lines were transfected with each of the corresponding intrabody expression plasmids separately using Lipofectamine 2000 to analyse expression of poly Sia and NCAM in these cells in the presence of the intrabodies (Fig. 5). The CHO celllines were used for this analysis because they express NCAM and ST8SiaII or ST8SiaVI in contrast to HEK293 cells which express only a very low amount of NCAM. They were also used for immunofluorescence to analyse the retention of PolySTs inside the ER by the intrabodies (Fig. 4).

After transient transfection of HEK293 cells with the DNA of PolySTs or DNAs of PolySTs and corresponding IBs cells were cultured for 48 h in DMEM, 10% heat inactivated FCS and pen/strep as antibiotics at standard concentrations under normal oxygen tension with 5% CO_2_ in a humified cell culture incubator (Heraus, Hanau, Germany).

Recombinant CHO cells after transient transfection with the DNA of the corresponding anti-polySTs IBs were cultured for 48 h in the same medium as HEK293 transfected cells. After stable triple transfection of TE671 cells with the anti-PolySTs IBs expression plasmids and luciferase expression plasmid cells were grown in DMEM Medium containing 10% FCS, pen/strep and 1000 μg/ml neomycin, 500 μg/ml zeocin and 1 μg/ml puromycin. TE671 cells stable transfected with the expression plasmid of the anti-NCAM intrabody were cultivated in DMEM Medium with 10% inactivated FCS, pen/strep and 0.4 mg/ml neomycin.

### Construction of IBs

Construction of anti-ST8SiaII-IB and anti-ST8SiaIV-IB was performed following the methodology described in [42]. RNA was purified from hybridoma cells and transcribed into cDNA by random priming. For adapter ligation the single stranded cDNA was converted into double stranded cDNA. After adapter ligation, cDNA encoding the variable Ig domains were amplified by PCR using primers complementary to the adapter and the sequence encoding the conserved constant domain.

The sequence information for the variable domains of heavy and light chain was obtained by sequencing and used for the generation of two pairs of sequence-specific primers. DNA fragments encoding V_H_ and H_L_ domain of anti-ST8SiaII mAb 3167 were amplified using the primer pairs VHBACK-SALI-STX3167 5’ CAACTgCAggTCgACCAggTCCAACTgCAgCAgCCTggg 3’/VHFOR-STX3167 5’ TgAggAgACTgTgAgAgTggTgCCTTg 3’ and VLBACK-STX3167 5’ gATgTTgTggTgACTCAAACTCCACTC 3’/VLFOR-NOTI-STX3167 5’ TTTgATgCggCCgCCCgTTTgATTTCCAgCTTggTgCC 3’. DNA fragments encoding V_H_ and H_L_ domain of anti-ST8SiaIV mAb 3175 were amplified using the primer pairs VHBACK-SALI-PST3175 5’ CAACTgCAggTCgACgAggTTCAgCTgCAgCAgTCTggg 3’/VHFOR-PST3175 5’ TgAggAgACggTgACTgAggTTCCTTg 3’ and VLBACK-PST3175 5’ AACATTATgATgACACAgTCgCCATCA 3’/VLFOR-NOTI-PST3175 5’ TTTgATgCggCCgCCCgTTTTATTTCCAgCTTggTCCC 3’. For assembly of the scFv-DNA a linker oligonucleotide was synthesized and used in an assembly-PCR (anti-ST8SiaII: LINKER-STX3167 5’ ggCACCACTCTCACAgTCTCCTCAggTggAggCggTTCAggCggAggTggCTCTggCggTggCggATCggATgTTgTggTgACTCAAACTCCA 3’; anti-ST8SiaIV: LINKER-PST3175 5’ggAACCTCAgTCACCgTCTCCTCAggTggAggCggTTCAggCggAggTggCTCTggCggTggCggATCgAACATTATgATgACACAgTCgCCA 3’. At last, the scFv-DNA was cloned into the expression vector pCMV/myc/ER. This leads to the expression plasmids for both anti-polyST’s IBs pCMVmycER/αST8SiaII-IB and pCMVmycER/αST8SiaVI-IB.

### Transient transfection

10^6^ HEK293 cells cultivated on a well of a 6 well microtiter plate were transiently transfected for 48 h with 4 μg DNA of ST8SiaII or 4 μg DNA of ST8SiaIV. In addition cotransfection of 2 μg ST8SiaII or 2 μg ST8SiaIV with 2 μg of the corresponding intrabody expression plasmid was performed. Recombinant CHO cells expressing ST8SiaII or ST8SiaIV were similarly transfected with the corresponding IBs. Transfection was performed with Lipofectamine 2000 according to supplier instructions (in vitro gene).

### Immunofluorescence analysis

Cells were fixed with 4% paraformaldehyde (10 min at room temperature) followed by permeabilization with 0.1% Triton X-100 for 10 min. Cells were washed three times with PBS/3%BSA and blocked for 20 min with the same solution. The endoplasmic reticulum was visualized using mouse anti-calnexin AF18 (Abcam) and goat anti-mouse Cy3 labeled antibody (Dianova), the Golgi apparatus was visualized using rabbit anti-α-Mannosidase II [83] and goat anti-rabbit Cy3 labeled antibody (Dianova). The IBs were detected with mouse anti-c-myc 9E10 (Santa Cruz Biotechnology) and goat anti-mouse Cy3 labeled antibody (Dianova) or directly with goat anti-c-myc FITC conjugated antibody (Novus Biologicals). Flag-HA-tagged polySTs were detected with mouse anti-FLAG M5 (Santa Cruz Biotechnology) and goat anti-mouse Cy3 or goat anti-mouse FITC labeled antibody. Primary antibodies were incubated for 2 h and secondary antibodies for 1 h (diluted in 3% BSA/PBS) at room temperature. In between, cells were washed 5 times with PBS-0.05%Tween. For analysis by laser scanning confocal microscopy, cells were coated with Vectashield Mounting Medium (Vector Laboratories).

### SDS-PAGE/Immunoblotting

A discontinuous SDS-PAGE was performed using 7% acrylamide gels for high molecular weight proteins (NCAM, polysialylated NCAM) and 10% for low molecular weight proteins (polySTs, IBs). Samples were incubated for 5 min at 95 °C or for 10 min at 65 °C if polySia was supposed to be detected. In this case an additional incubation step with endosialidase [84] was inserted for the negative control. Protein blotting was performed via semi-dry blot on a PVDF membrane using 48 mM Tris, 39 mM Glycerin in H_2_O. After blocking for 30 min with 5% skimmed milc detection of the PolySTs were performed with mouse anti-Flag M5 (Santa Cruz Biotechnology) and goat anti-mouse IgG Fc peroxidase labelled antibody (Jackson ImmunoResearch).

The transfected CHO cell lysates were directly analysed by immunoblotting with mouse anti-polySia 735 antibody [85] (polySia detection) and goat anti-mouse IgG Fc peroxidase labelled antibody (Jackson ImmunoResearch). NCAM was detected with rat anti-NCAM H28 antibody [86] and goat anti-rat peroxidase conjugated antibody (Santa Cruz Biotechnology).

Primary antibodies were incubated for 2 h and secondary antibodies for 1 h, diluted in 2% skimmed milc. Staining was performed with Sigma Fast 3,3’-Diaminobenzidin (DAB) Tetrahydrochlorid/Sigma Fast Metal Enhancer (Sigma Aldrich) according to supplier instructions.

### ELISA

Each purified soluble polyST was coated overnight on 96-well MaxiSorb™ polystyrene assay plates (Nunc) and detected with mouse monoclonal anti-ST8SiaII and anti-ST8SiaIV antibodies or the respective IBs (incubation time 2 h at room temperature). Blocking was performed with 100 μl 5% skimmed milk in PBS for 1 h at 37 °C. In case of the IB 1 × 10^6^ HEK293 cells (DMSZ) were transiently transfected with the expression plasmids using Lipofectamine 2000 (Invitrogen) and after 48 h lysed on ice for 30 min with 100 μl 50 mM Tris–HCl (pH 8.0), 1 mM MgCl_2_, 1% NP-40 in H_2_O. After centrifugation in a table top centrifuge a supernatant aliquot or the purified monoclonal antibodies were then applied in a serial dilution. An additional incubation step with 100 μl mouse anti-c-myc 9E10 (Santa Cruz Biotechnology) was inserted for the IBs (incubation time 1 h). Detection was performed using 100 μl goat anti-mouse HRP antibody (Jackson ImmunoResearch, incubation time 1 h). For development of the signals 50 μl tetramethylbenzidine (Sigma Aldrich) was added to each well after incubation with detection antibody and 5 times washing with PBS 0.5%Tween. The colour reaction was stopped with 50 μl 1 M H_2_SO_4_ and absorption red at 450 nm.

### Immunoprecipitation

Immunoprecipitation was performed with HEK 293 cells or recombinant CHO cell lines expressing one of the polySTs. HEK 293 cells were transfected by using Lipofectamine 2000 (Invitrogen) with the expression plasmids for ST8SiaII or ST8SiaIV or in addition co-transfected with the corresponding IB DNA to analyse the different glycosylated forms of the polySTs and the complex of polyST and corresponding IB.

After culturing of the HEK293 cells for 2 days they were lysed for 30 min on ice with 100 μl 50 mM Tris–HCl (pH 8), 1 mM MgCl_2_, 1% NP-40 in H_2_O and filled up with 900 μl PBS followed by an incubation with 2 μg of precipitating antibody for 1 h at room temperature. The polySTs from polySTs transfected HEK293 cells were precipitated with mouse anti-HA 12CA5 antibody and the polyST-IB complexes from polyST/IB cotransfected HEK293 cells with mouse-anti-myc antibody.

Afterwards the samples were mixed with 20 μl Protein G PLUS Agarose (Santa Cruz Biotechnology) at 4 °C overnight. After washing the agarose pellet 4 times with PBS the samples were used for PAGE and immunoblotting with mouse anti-FLAG M5 antibody and goat anti-mouse IgGFc peroxidase labelled antibody.

### Flow cytometry

Generated stable anti-polyST IBs and luciferase expressing TE671 cell lines were incubated with anti-polySia 735 [85] or mouse anti-NCAM ERIC1 (Santa Cruz Biotechnology) antibodies to determine polySia and NCAM surface expression by flow cytometry. Staining with antibodies was performed for 30 min at 4 °C in a 96-well microtitre plate (Nunclon™ Surface plate, Nunc) in 100 μl PBS containing 2% FCS (Invitrogen). For detection of the primary antibodies an goat anti-mouse RPE-labeled antibody (Dianova) was used. As negative controls an incubation step with endosialidase [84] before applying the primary detection antibody was performed or the primary anti-NCAM antibody was not used. Cells were washed once with PBS containing 2% FCS and resuspended in 300 μl PBS containing 2% FCS and 10 μg/ml propidiumiodide for subsequent analysis using a FACS-calibur equipped with CellQuest software (Becton Dickinson).

### Proliferation assay

Cells of each generated stable anti-polyST IBs and luciferase expressing TE671 cell line were cultured on 6-well plates. The number of cells per well was monitored every 24 h for 8 days (counting of living cells after staining the cells with trypan blue).

### Generation of stable ST8SiaII-IB and ST8SiaVI-IB expressing TE671 cells

To test the functionality of the IBs in vivo on metastasis of the rhabdomyosarcoma tumor cells in xenografted tumor mice, stable intrabody expressing TE671 cells were generated. For this purpose the αST8SiaII-IB coding DNA sequence was cloned into the vector pSV40/Zeo/intrabody A7 containing the ER intrabody scFv A7 [87] and the resistance gene Zeocin. The αST8SiaII-IB was inserted into the vector via *Sal*I and *Xba*I sites to ensure a different antibiotic resistance for selection. Furthermore, the myc detection tag was mutated into a HA tag by site-directed mutagenesis using the primers: MYCtoHA-BACK 5’ CgggCggCCgCATACCCATACgACgTCCCAgACTACgCTAATggAgCTgCAAgCgAgAAg 3’ and MYCtoHA-FOR 5’ CTTCTCgCTTgCAgCTCCATTAgCgTAgTCTgggACgTCgTATgggTATgCggCCgCCCg 3’. This leads to the expression vector pSV40/Zeo/αST8SiaII-IB.

TE671 cells expressing both polySTs were then transfected with both intrabody expression plasmids pCMVmycER/αST8SiaVI-IB and pSV40/Zeo/αST8SiaII-IB and stable clones were selected. As control cell line the empty vector pCMV/myc/ER was used as transfectant. All three cell lines were also transfected with the luciferase expression plasmid pVBC3luc2puro (A.Kröger, HZI) for later visualization in vivo. Additionally, an already existing intrabody against NCAM (αNCAM-IB) was applied [42] to compare the effect of the loss of polySia and the loss of NCAM cell surface expression.

### Mouse experiments

10^6^ cells in 100 μl PBS of each generated stable anti-polySTs IBs/luciferase or anti-NCAM intrabody/luciferase expressing TE671 cell line were injected intraperitonally (i.p.) into 20 weeks old C57BL/6 J RAG-2 mice lacking B-, T-, NK- and NKT cells. For detection of cells 100 μl D-luciferine potassium salt (33 mg/ml in PBS, Synchem) were injected i.p. and luminescence was measured every week via IVIS 200 and Living Image software (Perkin Elmer). As anaesthetic was used Isofluran. Isofluran was choosen because the depth of anaestesia can be easily regulated. All experiments were done in the laboratories of the Central Animal Facility at the HZI.

Housing was performed under SPF. Cage size: 502 cm^2^, bedding material: aspen with tissue paper as enrichement. 3 mice pro cage. Mice were sacrificed upon study completion by cervical dislocation.

## Additional files

Additional file 1: Binding of anti-ST8SiaII-IB and anti-ST8SiaIV-IB to their antigens analyzed in ELISA. Analysis was performed as in Fig. 2. Data from two independent experiments. Mean values and corresponding standard deviations are shown. (XLSX 23 kb)

Additional file 2: *﻿A*, ﻿Inhibitory effect of anti-ST8SiaII-IB and anti-ST8SiaIV-IB on metastasis of rhabdyomasarcoma cells after 4 weeks of tumor cell injection in mice. Mean values of each group of mice with corresponding standard deviation. Rhabdomyosarcoma cells expressing anti-ST8SiaII-IB and anti-ST8SiaIV-IB or anti-NCAM-IB or as negative control tumor cells stable transfected with the empty vector were injected intraperitoneally into 3 C57BL/6 J RAG-2 mice at a time. Luminiscence was determined at week 4 using in vivo imaging systems (IVIS). Shown are the results as luminiscence signals (p/sec/cm^2^/sr). ROI: region of interest. The red circles shows the metastic tumor cells in the region of lung and liver. *B*, Mean values of each group of mice with corresponding standard deviation. (XLS 1903 kb)

Additional file 3: Luminescence picture after injection of control tumor cells with empty vector in mice (week 4). Luminescence was determined at week 4 using in vivo imaging system (IVIS). On the ride side are seen the luminescence signals (p/Sec/cm^2^ /sr). (TIF 287 kb)

Additional file 4: Luminescence picture after injection of tumor cells expressing anti-ST8SiaII-IB and anti-ST8SiaIV-IB in mice (week 4). Luminescence was determined at week 4 using in vivo imaging system. On the ride side are seen the luminescence signals (p/Sec/cm^2^/sr). (TIF 286 kb)

Additional file 5: Luminescence picture after injection of tumor cells expressing anti-NCAM-IB in mice (week 4). Luminescence was determined at week 4 using in vivo imaging system. On the ride side are seen the luminescence signals (p/Sec/cm^2^/sr). (TIF 288 kb)

Additional file 6: Tumor growth tracking of rhabdyomasarcoma cells expressing the empty vector pCMV/myc/ER (A) or the anti-ST8SiaII-IB and anti-ST8SiaIV-IB (B) in mice over a period of six weeks. 10^6^ rhabdomyosarcoma cells in 100 μl PBS as negative control stable transfected with the empty vector pCMV/myc/ER (A) or expressing anti-ST8SiaII-IB and anti-ST8SiaIV-IB (B) were injected intraperitoneally into 3 C57BL/6 J RAG-2 mice at a time. Luminiscence was determined at week 1 to 6 using in vivo imaging systems (IVIS). Li = Luminiscence intensity. (TIF 960 kb)

Additional file 7: Tumor growth tracking of rhabdyomasarcoma cells expressing the anti-NCAM IB. 10^6^ rhabdomyosarcoma cells in 100 μl PBS expressing anti-NCAM-IB were injected intraperitoneally into 3 C57BL/6 J RAG-2 mice at a time. Luminiscence was determined as described in Additional file 6: Figure S1. (TIF 783 kb)

## Abbreviations

Co-IP: Co-Immunoprecipitation
ER IBs against the polySTs ST8SiaII and –IV: αST8SiaII-IB and αST8SiaIV-IB
IB: Intrabodies
NCAM: Neural cell adhesion molecule
Pen: Penicillin
PolySia: Polysialylic acid
PolyST: Polysialyltransferase
Strep: Streptomycin
