# Supplementary figures and images for: Intrabodies against the Polysialyltransferases ST8SiaII and ST8SiaIV inhibit Polysialylation of NCAM in rhabdomyosarcoma tumor cells
Source: BMC Biotechnol. 2017 May 12;17:42. doi: 10.1186/s12896-017-0360-7 (PMC5429572; doi:10.1186/s12896-017-0360-7)

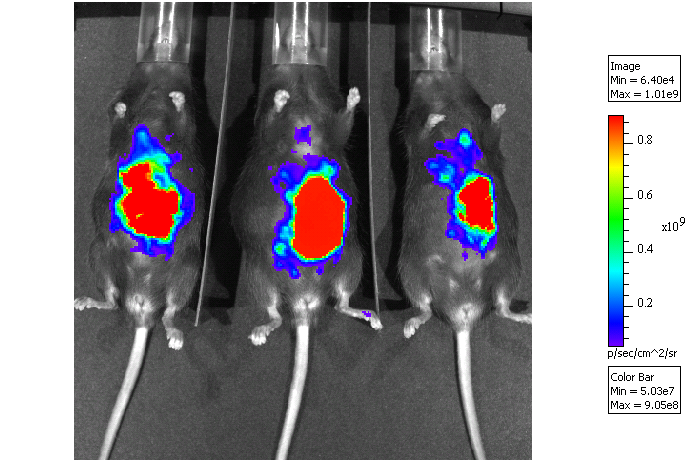

Supplement: Supplementary file 3 — Luminescence picture after injection of control tumor cells with empty vector in mice (week 4). Luminescence was determined at week 4 using in vivo imaging system (IVIS). On the ride side are seen the luminescence signals (p/Sec/cm2 /sr). (TIF 287 kb) [file 12896_2017_360_MOESM3_ESM.tif]

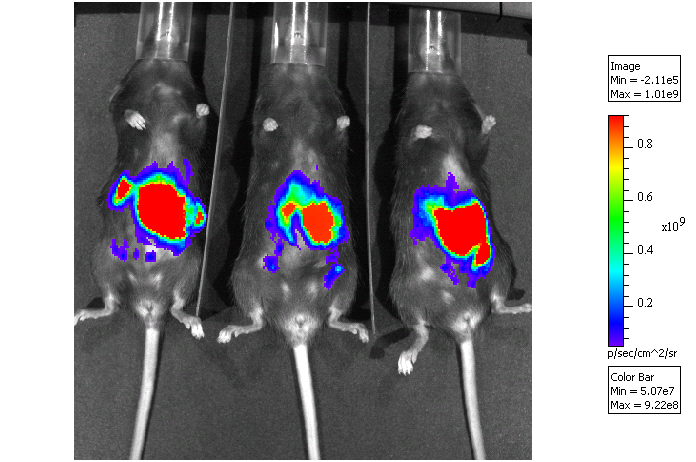

Supplement: Supplementary file 4 — Luminescence picture after injection of tumor cells expressing anti-ST8SiaII-IB and anti-ST8SiaIV-IB in mice (week 4). Luminescence was determined at week 4 using in vivo imaging system. On the ride side are seen the luminescence signals (p/Sec/cm2/sr). (TIF 286 kb) [file 12896_2017_360_MOESM4_ESM.tif]

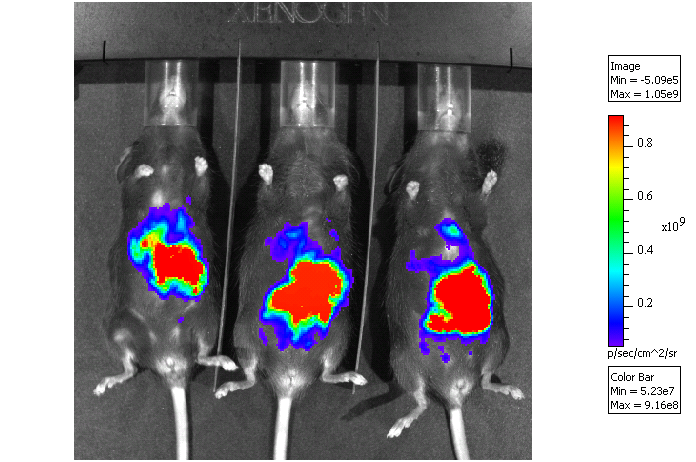

Supplement: Supplementary file 5 — Luminescence picture after injection of tumor cells expressing anti-NCAM-IB in mice (week 4). Luminescence was determined at week 4 using in vivo imaging system. On the ride side are seen the luminescence signals (p/Sec/cm2/sr). (TIF 288 kb) [file 12896_2017_360_MOESM5_ESM.tif]

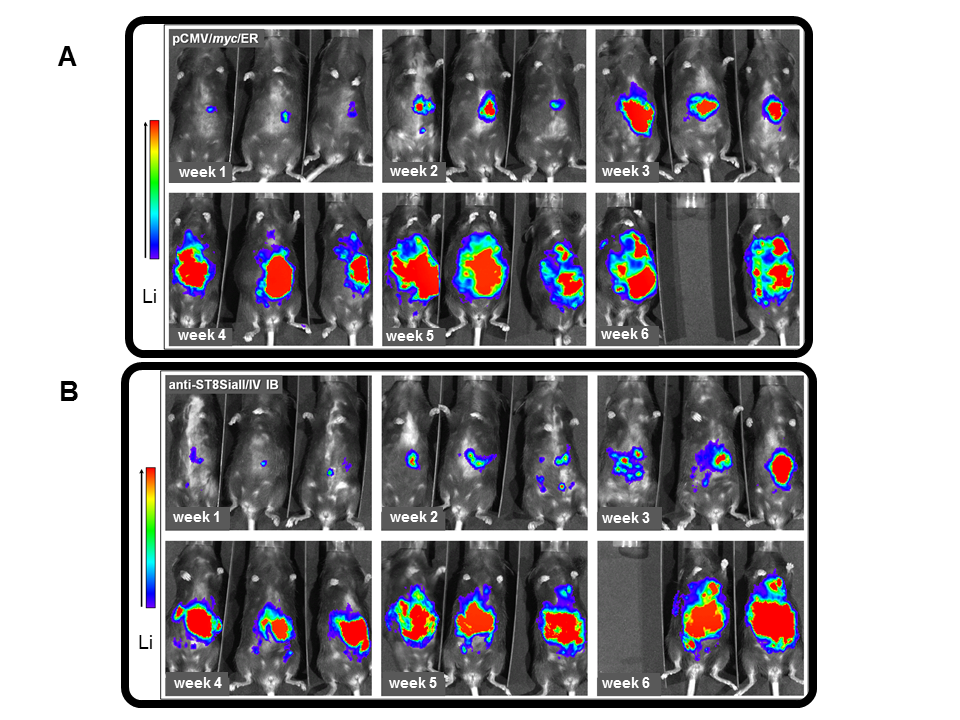

Supplement: Supplementary file 6 — Tumor growth tracking of rhabdyomasarcoma cells expressing the empty vector pCMV/myc/ER (A) or the anti-ST8SiaII-IB and anti-ST8SiaIV-IB (B) in mice over a period of six weeks. 106 rhabdomyosarcoma cells in 100 μl PBS as negative control stable transfected with the empty vector pCMV/myc/ER (A) or expressing anti-ST8SiaII-IB and anti-ST8SiaIV-IB (B) were injected intraperitoneally into 3 C57BL/6 J RAG-2 mice at a time. Luminiscence was determined at week 1 to 6 using in vivo imaging systems (IVIS). Li = Luminiscence intensity. (TIF 960 kb) [file 12896_2017_360_MOESM6_ESM.tif]

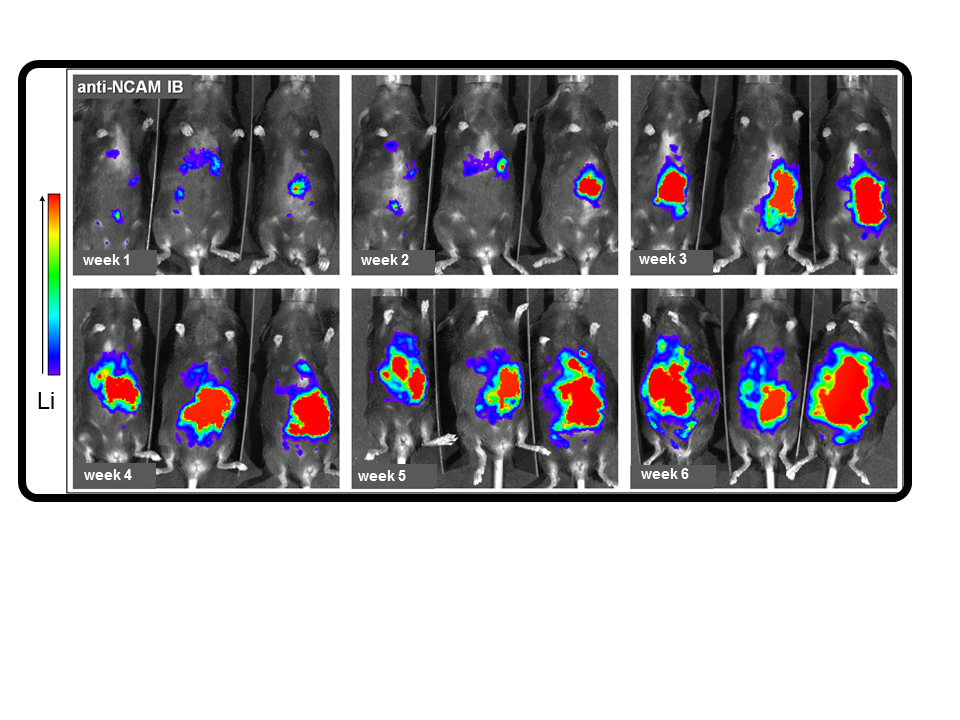

Supplement: Supplementary file 7 — Tumor growth tracking of rhabdyomasarcoma cells expressing the anti-NCAM IB. 106 rhabdomyosarcoma cells in 100 μl PBS expressing anti-NCAM-IB were injected intraperitoneally into 3 C57BL/6 J RAG-2 mice at a time. Luminiscence was determined as described in Additional file 6: Figure S1. (TIF 783 kb) [file 12896_2017_360_MOESM7_ESM.tif]
